# Comparative Stability and Anesthetic Evaluation of Holy Basil Essential Oil Formulated in SNEDDS and Microemulsion Systems in *Cyprinus carpio* var. Koi

**DOI:** 10.3390/pharmaceutics17080997

**Published:** 2025-07-31

**Authors:** Kantaporn Kheawfu, Chuda Chittasupho, Surachai Pikulkaew, Wasana Chaisri, Taepin Junmahasathien

**Affiliations:** 1 Faculty of Pharmacy, Chiang Mai University, Chiang Mai 50200, Thailand; kantaporn.kheawfu@cmu.ac.th (K.K.); chuda.c@cmu.ac.th (C.C.); 2 Faculty of Veterinary Medicine, Chiang Mai University, Chiang Mai 50100, Thailand; surachai.pikul@cmu.ac.th (S.P.); wasana.ch@cmu.ac.th (W.C.)

**Keywords:** holy basil essential oil, self-nanoemulsifying drug delivery system (SNEDDS), microemulsion, anesthetic activity, eugenol, aquaculture

## Abstract

**Background/Objectives:** Holy basil (*Ocimum tenuiflorum* L.) essential oil exhibits antioxidant, antimicrobial, and anesthetic activities, mainly due to eugenol, methyl eugenol, and β-caryophyllene. However, its clinical application is limited by poor water solubility, instability, and low bioavailability. This study developed and compared two delivery systems, self-nanoemulsifying drug delivery systems (SNEDDS) and microemulsions (ME), to enhance their stability and fish anesthetic efficacy. **Methods:** The optimized SNEDDS (25% basil oil, 8.33% coconut oil, 54.76% Tween 80, 11.91% PEG 400) and ME (12% basil oil, 32% Tween 80, 4% sorbitol, 12% ethanol, 40% water) were characterized for droplet size, PDI, zeta potential, pH, and viscosity. Stability was evaluated by monitoring droplet size and PDI over time and by determining the retention of eugenol, methyl eugenol, and β-caryophyllene after storage at 45 °C. Fish anesthetic efficacy was tested in koi carp (*Cyprinus carpio* var. koi). **Results:** SNEDDS maintained a small droplet size (~22.78 ± 1.99 nm) and low PDI (0.188 ± 0.088 at day 60), while ME showed significant size enlargement (up to 177.10 ± 47.50 nm) and high PDI (>0.5). After 90 days at 45 °C, SNEDDS retained 94.45% eugenol, 94.08% methyl eugenol, and 88.55% β-caryophyllene, while ME preserved 104.76%, 103.53%, and 94.47%, respectively. In vivo testing showed that SNEDDS achieved faster anesthesia (114.70 ± 24.80 s at 120 ppm) and shorter recovery (379.60 ± 15.61 s) than ME (134.90 ± 4.70 s; 473.80 ± 16.94 s). Ethanol failed to induce anesthesia at 40 ppm and performed poorly compared to SNEDDS and ME at other concentrations (*p* < 0.0001). **Conclusions:** SNEDDS demonstrated superior physical stability and fish anesthetic performance compared to ME. These findings support SNEDDS as a promising formulation for delivering holy basil essential oil in biomedical and aquaculture applications.

## 1. Introduction

Holy basil (*Ocimum tenuiflorum* L.) is a widely used medicinal herb in traditional and modern herbal therapies due to its broad spectrum of pharmacological activities, including antioxidant, anti-inflammatory, antimicrobial, and adaptogenic properties [1,2,3,4]. The therapeutic potential of holy basil essential oil is largely attributed to its volatile constituents, such as eugenol, methyl eugenol, and β-caryophyllene. However, the clinical application of essential oil-based extracts is often hindered by poor solubility, instability, and low bioavailability [5].

However, despite their promising bioactivity, few studies have explored the use of nanoformulations of essential oils specifically for fish anesthesia, representing a significant gap in the current literature. To overcome these limitations, lipid-based nanocarrier systems such as self-nanoemulsifying drug delivery systems (SNEDDS) and microemulsions (ME) have gained increasing attention [6,7]. These systems enhance the solubilization of lipophilic phytoconstituents, improve physicochemical stability, and promote absorption through mucosal and intestinal membranes [8]. SNEDDS are isotropic mixtures of oils, surfactants, and co-surfactants that spontaneously form oil-in-water nanoemulsions upon contact with aqueous fluids [9]. In contrast, ME are thermodynamically stable dispersions formed with water, oil, and surfactant systems, typically resulting in translucent, low-viscosity formulations [10].

Despite the advantages of both systems, their physical and chemical stabilities under various storage conditions remain critical for practical formulation development. Parameters such as particle size, polydispersity index (PDI), zeta potential, viscosity, and pH are essential indicators of formulation performance over time [11]. Variations in aqueous content and the impact of temperature fluctuations can significantly affect formulation stability. In aquaculture, the use of anesthetic agents is essential for reducing stress, injury, and mortality during routine handling, transport, vaccination, or invasive procedures. Synthetic anesthetics such as MS-222, benzocaine, or clove oil derivatives are widely used; however, they may pose risks related to environmental persistence, residual toxicity, and potential adverse effects on fish health or consumer safety [12]. Consequently, there is growing interest in exploring natural, plant-derived alternatives with anesthetic potential. Essential oils, particularly those containing eugenol and related compounds, offer promising bioactivity with fewer ecological and toxicological concerns. Their rapid action and biodegradability make them attractive candidates for replacing or complementing conventional anesthetics in sustainable aquaculture practices [13,14].

Koi carp (*Cyprinus carpio* var. koi), a brightly colored variant of the common carp, is among the most prominent groups of freshwater ornamental fish globally. Valued for their striking coloration and distinctive body form, koi carp are widely cultivated as ornamental pets. As a result, their commercial aquaculture has become a significant economic contributor to the pet industry and continues to play a growing role in supplying the global market for ornamental fish enthusiasts [15].

However, the application of essential oils in aquaculture remains limited by formulation challenges, including low solubility in water and variable absorption across the gill membranes. Advanced delivery systems such as SNEDDS and ME may overcome these obstacles by enhancing the dispersion, solubilization, and bioavailability of essential oil constituents. By facilitating efficient absorption, these systems have the potential to improve the onset, depth, and consistency of anesthesia in fish while minimizing formulation-related limitations.

Therefore, this study hypothesizes that SNEDDS and ME formulations of holy basil essential oil can enhance anesthetic efficacy in koi carp compared to conventional ethanolic preparations, by improving the solubilization, stability, and bioavailability of active components such as eugenol and methyl eugenol. This hypothesis was tested through physicochemical characterization, stability studies, and in vivo anesthetic evaluation, aiming to establish a more effective and sustainable approach to fish sedation using natural bioactive compounds.

## 2. Materials

Holy basil (*Ocimum tenuiflorum* L.) essential oil was obtained from Thai China Flavors and Fragrances Industry Co., Ltd. (Bangkok, Thailand). The essential oil was verified to contain eugenol, methyl eugenol, and β-caryophyllene as its major constituents, based on GC-MS profiling analyzed by the Science and Technology Service Center, Faculty of Science, Chiang Mai University (STSC-CMU). Polyethylene glycol 400 (PEG 400), Tween 80, sorbitol, refined coconut oil, and ethanol were purchased from Namsiang Co., Ltd. (Bangkok, Thailand). Acetonitrile was procured from RCI Labscan Limited. (Bangkok, Thailand). Eugenol, methyl eugenol, and β-caryophyllene were purchased from Sigma-Aldrich (St. Louis, MA, USA)

## 3. Methods

### 3.1. Preparation of Holy Basil SNEDDS

The holy basil SNEDDS was formulated using the microemulsification technique, and the final formulation was produced without the use of water. The components were prepared as follows (*w*/*w*): 54.76 g of Tween 80 (surfactant), 11.91 g of polyethylene glycol (PEG) 400 (co-surfactant), 25.00 g of basil essential oil (active ingredient), and 8.33 g of refined coconut oil (carrier oil), making a total of 100 g. Initially, Tween 80 and PEG 400 were accurately weighed and combined in a single beaker. Polyethylene glycol 400 (PEG 400) was selected as a co-surfactant in the SNEDDS formulation due to its amphiphilic nature, which facilitates the solubilization of lipophilic essential oils and contributes to spontaneous nanoemulsion formation upon aqueous dilution. The mixture was manually stirred at ambient temperature until a clear and homogeneous solution was obtained. Subsequently, 25.00 g of basil essential oil and 8.33 g of refined coconut oil were weighed and added to the surfactant mixture. The resulting mixture was stirred continuously until a uniform and visually homogeneous formulation was achieved. The final SNEDDS formulation was stored in a tightly closed amber glass container at room temperature until further analysis.

### 3.2. Preparation of Holy Basil ME

**Figure 1:**
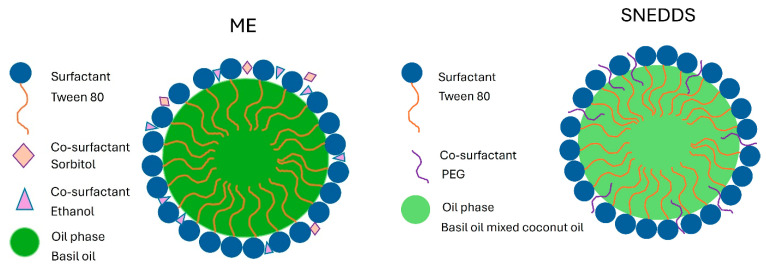
Schematic representation of the formulation structure of the microemulsion (ME) and self-nanoemulsifying drug delivery system (SNEDDS) loaded with basil oil.

The holy basil ME was prepared using a phase titration method based on a pre-concentrate formulation. The formulation consisted of 12 g of basil essential oil (oil phase), 32 g of Tween 80 (surfactant), 4 g of sorbitol (co-surfactant), 12 g of ethanol (co-surfactant), and 40 g of distilled water, totaling 100 g. Sorbitol and ethanol were selected based on prior reports and preliminary optimization, where their combined use enhanced the solubilization of the oil phase and promoted the formation of a stable, transparent microemulsion. First, 12 g of holy basil essential oil was weighed and transferred into a beaker to serve as the oil phase. Tween 80 (32 g) and sorbitol (4 g) were subsequently added to the oil phase. The mixture was stirred using a magnetic stirrer (IKA-Werke GmbH & Co. KG, Staufen, Germany) at room temperature until a homogeneous solution was observed. Then, 12 g of ethanol was added and mixed thoroughly. Ethanol was included as a co-surfactant to enhance solubilization of the oil phase and reduce interfacial tension, thereby supporting the formation and stabilization of the microemulsion system. Distilled water (40 g) was gradually added in small portions under continuous magnetic stirring. The addition of water was carried out slowly to avoid phase separation and ensure uniform mixing. The formation of a clear or slightly translucent solution indicated the successful development of an ME system. The final formulation was stored in a tightly sealed amber glass container at room temperature until further analysis. To illustrate the compositional and structural differences between the two delivery systems, Figure 1 presents a schematic comparison of the microemulsion (ME) and self-nanoemulsifying drug delivery system (SNEDDS) containing basil oil.

### 3.3. Characterization of Holy Basil SNEDDS and ME

The holy basil SNEDDS and ME formulations were characterized for their droplet size, PDI, and zeta potential using dynamic light scattering (DLS) at a scattering angle of 173° and a temperature of 25 °C (Zetasizer Nano Series, Malvern Instruments, Malvern, UK). The 173° angle was selected as it is the default backscattering angle for the instrument’s non-invasive backscatter (NIBS) technology, which minimizes multiple scattering effects and improves accuracy for small particle size measurements in low-volume, concentrated, or optically dense samples.

### 3.4. Determination of Physical Stability of Holy Basil SNEDDS and ME

The physical stability of the holy basil SNEDDS and ME formulations was evaluated under different temperature conditions over a 90-day period. Formulations were placed in tightly sealed amber glass containers and stored at 4 °C, 30 °C, and 45 °C at intervals of 0, 14, 30, 60, and 90 days. The hydrodynamic diameter, PDI, and zeta potential were measured at a freshly prepared concentration of 400 ppm of holy basil oil in all formulations, using the dynamic light scattering technique (Zetasizer ZS, Malvern Instruments, Malvern, UK) to monitor changes in particle size, PDI, and surface charge during storage. All samples were determined in triplicate.

### 3.5. Viscosity Measurement of Holy Basil SNEDDS and ME

The viscosity of holy basil SNEDDS and ME formulations was evaluated using a Brookfield rheometer (AMETEK Brookfield, Middleborough, MA, USA) equipped with a plate and plate geometry. The measurements were conducted at varying shear rates, ranging from 200 to 1000 s^−1^ for samples at room temperature. All samples were determined in triplicate.

### 3.6. pH Measurement of Holy Basil SNEDDS and ME

The pH of holy basil SNEDDS and ME formulations was diluted to 400 ppm and measured in triplicate at 25 °C using a calibrated pH meter (Thermo Fisher Scientific, Waltham, MA, USA).

### 3.7. Chemical Stability Study of Holy Basil SNEDDS and ME Formulations

The chemical stability of the major volatile constituents, eugenol, methyl eugenol, and β-caryophyllene, was investigated in both holy basil SNEDDS and ME formulations. Formulations were stored under three different conditions, refrigerated (4 ± 2 °C), room temperature (30 ± 2 °C), and accelerated (45 ± 2 °C), for a period of 90 days. Aliquots were collected on days 0, 14, 30, 60, and 90, and the residual content of each compound was quantified.

Quantitative analysis was performed using gas chromatography equipped with a flame ionization detector (GC-FID) by Agilent GC 7890B coupled with a PAL autosampler (RSI 85, CTC Analytics, Zwingen, Switzerland). The GC-FID method was adapted from a previously validated method described by Joy et al. [16], with modifications including the use of a different GC system (Agilent 7890B with PAL RSI autosampler, Agilent Technologies, Inc., Santa Clara, CA, USA), a reduced helium carrier gas pressure (9.92 psi), and adjusted FID gas flow settings (hydrogen: 30 mL/min, air: 400 mL/min, nitrogen: 25 mL/min). Samples were prepared by dissolving the formulation in acetonitrile to achieve a final concentration of holy basil oil of approximately 2 mg/mL. A 1 μL volume of sample was injected using a single taper split liner (Agilent Cat No: 19091J-413, Agilent Technologies, Inc., Santa Clara, CA, USA) with a split ratio of 50:1. All injections were performed in triplicate.

The separation was performed on an HP-5 capillary column (30 m × 0.32 mm i.d., 0.25 μm film thickness; 5% phenyl methylpolysiloxane; J&W Scientific, Folsom, CA, USA) using ultrahigh-purity helium as the carrier gas at a constant pressure of 9.92 psi. The injector and FID temperatures were maintained at 250 °C. The FID was supplied with ultrahigh-purity hydrogen (30 mL/min), scientific-grade air (400 mL/min), and nitrogen makeup gas (25 mL/min). The oven temperature program was as follows: initial temperature 50 °C held for 5 min, ramped at 3 °C/min to 120 °C, then at 5 °C/min to 250 °C, and finally at 15 °C/min to 300 °C with a final hold of 5 min, totaling 62.67 min run time.

### 3.8. In Vivo Anesthetic Activity of Holy Basil Formulations

The in vivo anesthetic activity of *Ocimum sanctum* (holy basil) essential oil formulated in SNEDDS, ME, and ethanol was evaluated in koi carp (*Cyprinus carpio* var. koi). Juvenile koi carp (weight: 301.6 ± 11.6 g and length: 13.2 ± 0.5 cm) were purchased from an ornamental fish shop in Chiang Mai, Thailand. During the acclimatization period, fish were maintained in 300 L plastic tanks filled with dechlorinated tap water. The water quality was controlled within the following parameters: pH 7.4–7.8, total hardness 122 ppm, alkalinity 110 ppm, and non-detectable levels of total ammonia nitrogen and nitrite. Approximately 30–50% of the water volume was replaced daily with fresh dechlorinated water. The fish were provided with a commercial pelleted feed containing 30% crude protein (Tetra Werke, Melle, Germany) once daily and kept under a natural photoperiod. After a quarantine period of two to four weeks, the fish were fasted for 12 h prior to transfer into glass tanks for anesthesia induction and recovery experiments.

All experimental procedures were conducted in accordance with the ethical guidelines approved by the Animal Care and Committee of the Faculty of Veterinary Medicine, Chiang Mai University (FVM-ACUC; Approval No. R6/2567, approved on 26 March 2024).

Each formulation was tested at final concentrations of 40, 60, 80, 100, and 120 ppm. The test solutions were freshly prepared by diluting the essential oil in each respective carrier. Ethanol in the highest dose (0.3% *v*/*v*) was used as the negative control. A total of 180 healthy koi carp were randomly divided into 18 groups (*n* = 10 per group), corresponding to each concentration and formulation type, including the ethanol control. Individual fish were transferred into 1 L glass aquaria containing 1 L of dechlorinated tap water with the designated concentration of test solution. The induction time to reach the surgical stage of anesthesia (Stage I3) was recorded. Anesthetic depth was evaluated based on behavioral criteria adapted from Ross and Ross [17]. Stage I1 was characterized by a partial loss of equilibrium, during which the fish exhibited rolling behavior and attempted to right itself. Stage I2 was defined as a total loss of equilibrium accompanied by a marked decrease in opercular ventilation. Stage I3 indicated deep anesthesia, evidenced by the absence of response to strong external stimuli. Confirmation of Stage I3 was performed by applying firm pressure near the caudal fin using forceps to verify the absence of a reflex response [11]. If Stage I3 was not achieved within 20 min, the test was terminated. Once the fish reached the surgical stage, it was immediately transferred to an anesthetic-free recovery tank containing clean dechlorinated water, and the recovery time was recorded as the time until resumption of normal swimming. The recovery process in fish was categorized into three distinct stages based on observable behavioral parameters: initial resumption of movement (Stage R1), restoration of regular opercular ventilation (Stage R2), and complete recovery of equilibrium and normal swimming posture (Stage R3) [18]. Each fish was tested only once.

### 3.9. Statistical Analysis

Statistical analysis was performed using one-way analysis of variance (ANOVA), followed by Tukey’s multiple comparisons as a post-hoc test to determine the significance of differences (GraphPad Prism version 7.02, La Jolla, CA, USA). A *p*-value of less than 0.05 was considered statistically significant. All data are expressed as mean ± standard deviation (SD) from three independent experiments (*n* = 3).

## 4. Results and Discussion

### 4.1. Effect of Storage Temperature on the Particle Size of Holy Basil SNEDDS and Holy Basil ME

**Figure 2:**
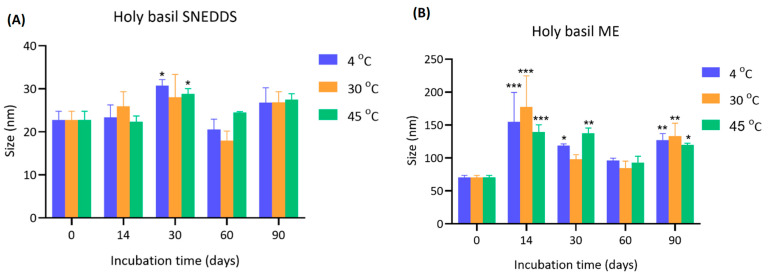
Particle size of (**A**) holy basil SNEDDS and (**B**) holy basil ME during storage at 4 °C, 30 °C, and 45 °C over a 90-day period. *, **, and *** indicate *p* < 0.05, *p* < 0.01, and *p* < 0.001, respectively.

The initial particle size of holy basil SNEDDS at day 0 was consistent across all temperatures (22.8 ± 2.0 nm). Over 90 days, minor fluctuations in size were observed. Notably, after 14 and 30 days, storage at 30 °C showed a slight increase in particle size (26.0 ± 3.4 nm and 28.0 ± 5.3 nm, respectively) compared to 4 °C and 45 °C. Statistically significant increases (* *p* < 0.05) were observed at day 30 for both 30 °C and 45 °C compared to 4 °C. Beyond 30 days, the particle size showed slight reductions or stable, with final measurements at day 90 ranging from 26.8 ± 3.5 nm to 27.5 ± 1.4 nm across all groups (Figure 2 and Supplementary Table S1).

For holy basil ME, the particle size was larger than holy basil SNEDDS. At day 0, the initial size was approximately 70.50 ± 3.15 nm at all temperatures. After 14 days, there was a marked and significant enlargement across all temperatures, especially at 30 °C (177.10 ± 47.50 nm), followed by 4 °C (154.87 ± 44.66 nm) and 45 °C (138.97 ± 11.26 nm). The increase was highly significant (*** *p* < 0.001) compared to day 0. The particle size remained elevated throughout the study, although a slight reduction was observed after 30 days. At day 90, the particle sizes were still significantly higher than initial values, with sizes of 127.27 ± 9.67 nm (4 °C), 133.13 ± 19.63 nm (30 °C), and 119.43 ± 2.98 nm (45 °C).

This study demonstrates that storage temperature and type of drug delivery system critically influence the particle size stability of the holy basil delivery system. Holy basil SNEDDS particle sizes remained relatively stable over 90 days, with only a minor increase observed at 14 and 30 days, particularly under 30 °C and 45 °C. These findings suggest that holy basil SNEDDS exhibit good physical stability across a range of storage temperatures. The minor increase observed at intermediate time points may be attributed to slight aggregation due to temporary thermal effects or surface energy changes. The stability at elevated temperatures highlights the robustness of holy basil SNEDDS. Conversely, the size of the holy basil ME was not stabilized, probably due to aggregation or coalescence, which was particularly noticeable after 14 days. The effect was most pronounced at 30 °C, indicating that moderate heating accelerated aggregation in aqueous environments, possibly due to the alteration of surfactant ability to surround hydrophobic droplets in the emulsion system, which enhanced molecular motion and interparticle interactions. Interestingly, even storage at 4 °C, a typically protective temperature, could not fully prevent particle growth when water was present, although it delayed the extent compared to 30 °C and 45 °C. This trend suggests that water in microemulsion facilitates hydration-mediated aggregation processes that override the stabilizing effects of low temperature. Additionally, the results imply that storage of holy basil SNEDDS without water in the formulation is preferable for maintaining the ability of the delivery system to provide stable holy basil nanoparticle size, especially at lower or moderate temperatures. When water is present, careful consideration of storage conditions is critical to mitigate aggregation and size enlargement, which could affect the functional properties and shelf-life of holy basil formulations.

### 4.2. Polydispersity Index of Holy Basil SNEDDS and ME Stored Under Different Conditions

**Figure 3:**
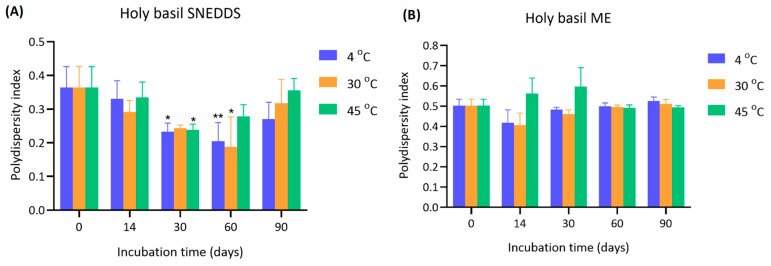
Polydispersity index (PDI) of (**A**) holy basil SNEDDS and (**B**) holy basil ME stored at 4 °C, 30 °C, and 45 °C over a 90-day period. Measurements were conducted at days 0, 14, 30, 60, and 90. * and ** indicate *p* < 0.05 and *p* < 0.01, respectively.

The PDI of holy basil SNEDDS was evaluated during storage for 90 days under different temperatures (4 °C, 30 °C, and 45 °C) (Figure 3). For holy basil SNEDDS, the initial PDI values at day 0 were around 0.364 ± 0.062. Over time, a significant decrease in PDI was observed at 30 and 60 days. At 30 days, PDI decreased to 0.234 ± 0.026 (4 °C), 0.244 ± 0.009 (30 °C), and 0.239 ± 0.017 (45 °C), showing a statistically significant reduction compared to day 0. However, at day 90, a slight increase in PDI was observed at all storage temperatures, returning toward initial values but still remaining lower than baseline. The initial PDI of holy basil ME was higher compared to holy basil SNEDDS (0.502 ± 0.032 at day 0). At day 30, the PDI values remained elevated, ranging from 0.461 ± 0.021 (30 °C) to 0.598 ± 0.093 (45 °C). At 90 days, the PDI remained high across all groups (e.g., 0.526 ± 0.019 at 4 °C). In contrast, in holy basil microemulsion, PDI remained high throughout the entire storage period, indicating persistent heterogeneity, which might be due to the alteration of surfactant capability leading to an increase in the opportunity for droplet fusion, coalescence, and dynamic instability. This suggests thermally induced droplet aggregation or coalescence due to surfactant reorganization within the microemulsion system. PDI values, especially at elevated temperatures like 45 °C, imply an increased risk of size growth and instability.

### 4.3. Zeta Potential of Holy Basil SNEDDS and ME During Storage

**Figure 4:**
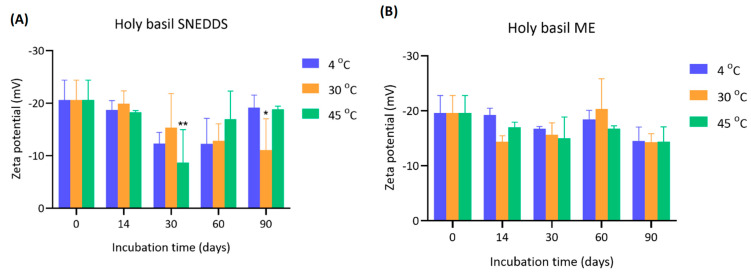
Zeta potential of (**A**) holy basil SNEDDS and (**B**) holy basil ME measured at 4 °C, 30 °C, and 45 °C over a 90-day storage period. * and ** indicate *p* < 0.05 and *p* < 0.01, respectively.

The zeta potential values of holy basil SNEDDS and ME were measured over 90 days under various storage temperatures (4 °C, 30 °C, and 45 °C) (Figure 4). The initial zeta potential of holy basil SNEDDS at day 0 was around −20.6 ± 3.80 mV for all groups. Over the incubation period, the magnitude of the zeta potential decreased, indicating a possibility in colloidal stability reduction. At 30 days, the zeta potential significantly shifted towards less negative values, especially at 30 °C (−15.37 ± 6.46 mV) and 45 °C (−8.70 ± 6.27 mV), with the 45 °C group exhibiting the most notable decrease (* *p* < 0.01 compared to day 0). These results also related to the temporary increase in particle size at 30 days. Similarly, at 90 days, a further significant decrease was observed in the 30 °C group (−11.07 ± 5.97 mV, * *p* < 0.05). Interestingly, samples stored at 4 °C maintained a relatively higher negative zeta potential across the time points, suggesting better stability under cold storage. The initial zeta potential of ME was approximately −19.60 ± 3.18 mV. Throughout the 90 days, the reduction in zeta potential was less dramatic compared to the SNEDDS. Slight fluctuations were observed at each time point, but no statistically significant differences were found. Final zeta potential values of ME remained within the range of −14 to −20 mV across all temperatures, with the highest reduction again observed at 45 °C at day 90 (−14.30 ± 2.74 mV). The overall trend suggests that the presence of water in ME slightly moderated the decline in zeta potential during storage compared to the holy basil SNEDDS.

A significant reduction in the magnitude of zeta potential was observed over time, particularly at elevated temperatures (30 °C and 45 °C), reflecting diminished electrostatic repulsion between droplets. It is well established that colloidal systems with zeta potentials below ±20 mV tend to be unstable due to insufficient repulsive forces to prevent aggregation [19]. The most severe loss of zeta potential at 45 °C suggests that high temperatures may disrupt the interfacial stability provided by the emulsifier, causing a decrease in the ability to strengthen the film surrounding the interfacial structure of the SNEDDS droplets, leading to reduced surface charge and induced aggregation. On the other hand, storage at 4 °C helped maintain a stronger negative surface charge over time, implying greater formulation stability at low temperatures. In the ME, although the zeta potential also decreased, the extent of reduction was more moderate and not statistically significant. However, even under aqueous conditions, the relatively low zeta potentials recorded at 90 days still indicate potential risks for droplet coalescence and aggregation if storage is prolonged. Taken together, these results demonstrate that both temperature and water presence in the formulation influence the electrokinetic stability of holy basil nano-formulations. Storage at low temperatures (4 °C) and minimizing water exposure are crucial for maintaining the negative zeta potential and ensuring long-term stability of the formulation. Special care must be taken during storage at elevated temperatures, especially without water, to prevent premature destabilization. However, in this study, Tween 80 was used as the main surfactant. Tween 80 is a non-ionic surfactant. Therefore, zeta potential may not be the only principal factor in stability. As can be seen in the SNEDDS particle size, although the zeta potential was significantly decreased, the particle size remained stable within 90 days.

### 4.4. pH Stability of Holy Basil SNEDDS and ME During Storage

**Figure 5:**
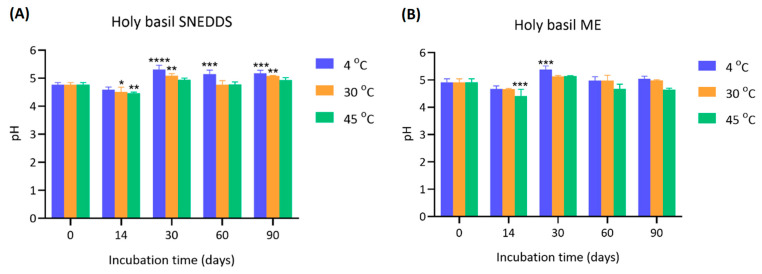
pH values of (**A**) holy basil SNEDDS and (**B**) holy basil ME stored at 4 °C (blue), 30 °C (orange), and 45 °C (green) over a 90-day period. *, **, *** and **** indicate *p* < 0.05, *p* < 0.01, *p* < 0.001 and *p* < 0.0001, respectively.

The pH values of holy basil SNEDDS and ME were measured at 0, 14, 30, 60, and 90 days under different storage temperatures (4 °C, 30 °C, and 45 °C) (Figure 5).

The initial pH values of SNEDDS at day 0 were approximately 4.77 ± 0.07 across all groups. After 14 days, a slight but significant increase in pH was observed, particularly in the 30 °C and 45 °C groups (* *p* < 0.05 and ** *p* < 0.01, respectively). The pH further increased markedly by day 30 across all storage temperatures, reaching 5.31 ± 0.15 (4 °C), 5.09 ± 0.07 (30 °C), and 4.94 ± 0.06 (45 °C), with highly significant differences compared to day 0 (*** *p* < 0.001 or **** *p* < 0.0001). The elevated pH levels were generally maintained through 60 and 90 days, with slight variations, but remained significantly higher than baseline. The initial pH of the ME was slightly higher than SNEDDS (4.92 ± 0.12). Over the incubation period, minor fluctuations were observed. At day 14, a slight but significant decrease in pH was detected in the 45 °C group (*** *p* < 0.001), while the other groups showed more stability. At day 30, a notable increase in pH was observed in the 4 °C group (5.38 ± 0.13, *** *p* < 0.001 compared to day 0), whereas samples stored at 30 °C and 45 °C showed more modest changes. After 60 and 90 days, the pH values of all groups stabilized, ranging from 4.97 to 5.15, with no drastic variations compared to earlier measurements.

A gradual and significant increase in pH was observed in SNEDDS, especially at 4 °C and 30 °C. The more stable increase observed at 4 °C and 30 °C compared to 45 °C suggests that milder storage temperatures promote a more controlled chemical transition, potentially due to slower oxidation reaction [20,21,22]. The ability of the formulation to maintain an elevated yet stable pH over 90 days further supports its chemical resilience in dry conditions. Conversely, in ME, the pH was relatively stable over time with smaller fluctuations. Water likely acts as a buffering medium, moderating changes in ionization and limiting significant pH shifts. However, at an elevated temperature (45 °C), a significant initial decrease in pH was detected. Nevertheless, stabilization of pH at later time points indicates that the formulation reached a new equilibrium, minimizing further changes. The data suggest that holy basil nano-formulations are more chemically stable in SNEDDS, particularly at moderate temperatures. These findings highlight the importance of minimizing water exposure and maintaining appropriate storage temperatures to ensure pH stability and, by extension, the overall integrity of the holy basil SNEDDS during long-term storage.

### 4.5. Viscosity of Holy Basil SNEDDS and ME During Storage

**Figure 6:**
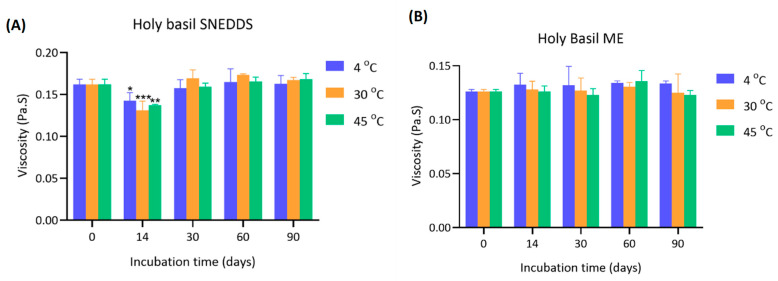
Viscosity of (**A**) holy basil SNEDDS and (**B**) holy basil ME during storage at 4 °C (blue), 30 °C (orange), and 45 °C (green) over 90 days. *, **, and *** indicate *p* < 0.05, *p* < 0.01, and *p* < 0.001, respectively.

The viscosity of holy basil SNEDDS was monitored over 90 days at different storage temperatures (4 °C, 30 °C, and 45 °C) in both holy basil SNEDDS and ME (Figure 6). The initial viscosity of SNEDDS at day 0 was approximately 0.162 ± 0.006 Pa·s across all groups. At day 14, a significant reduction in viscosity was observed, particularly in the 30 °C and 45 °C groups, where viscosity values decreased to 0.131 ± 0.011 and 0.137 ± 0.001 Pa·s, respectively (* *p* < 0.05 and **** *p* < 0.0001). After this initial decline, viscosity values gradually recovered by day 30 and remained stable through day 90, with final viscosities ranging from 0.162 to 0.168 Pa·s across all groups. In ME, the initial viscosity at day 0 was approximately 0.126 ± 0.002 Pa·s across all groups. Over the course of 90 days, the viscosity values remained relatively stable with only slight fluctuations. Minor reductions were observed at 30 and 90 days; however, no statistically significant changes were detected compared to day 0. Viscosity values at the end of storage ranged from 0.123 to 0.136 Pa·s, indicating good stability under aqueous conditions.

A significant but transient reduction in viscosity of SNEDDS was observed during the first 14 days, especially at higher storage temperatures (30 °C and 45 °C). This initial drop suggests that thermal effects may temporarily disrupt the homogeneity of the compositions or surfactant arrangement within the SNEDDS. This might be due to the coconut oil, which has a melting point of approximately 24–25 °C. The incubation period covered the congealing point, which might alter the viscosity of coconut oil, affecting the viscosity of formulations at the first measurement time point. This phenomenon demonstrates the self-stabilizing capacity of the holy basil SNEDDS system without water in the formulation. In ME, viscosity remained relatively stable throughout the 90-day period, with no significant changes. Water likely acted as a plasticizer or a stabilizing medium, moderating internal molecular mobility and minimizing structural disruption. The consistently low viscosity observed suggests that holy basil ME can maintain fluidity and homogeneity in aqueous environments over extended storage periods. Therefore, these findings highlight that while holy basil SNEDDS at elevated temperatures can transiently affect viscosity, the holy basil SNEDDS system is capable of structural recovery and homogeneity of the system.

### 4.6. Chemical Stability of SNEDDS and ME

The stability of eugenol, methyl eugenol, and β-caryophyllene in both SNEDDS and microemulsion (ME) formulations was evaluated under various storage conditions (4 °C, 30 °C, and 45 °C) for a period of 90 days. In general, all three compounds remained relatively stable throughout the study, with some variability observed depending on the formulation type and temperature.

**Figure 7:**
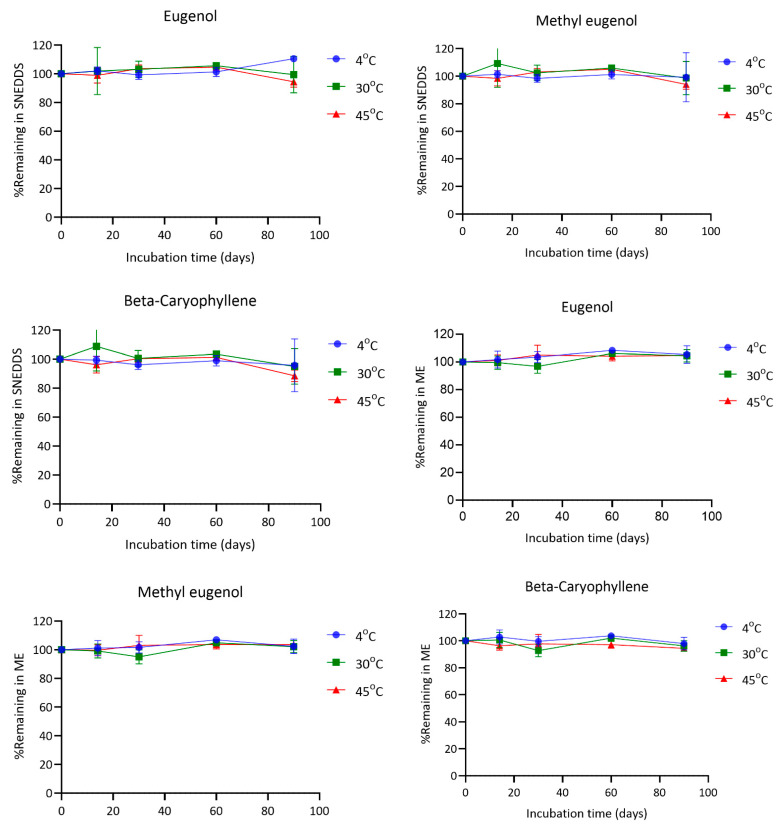
Stability profiles of major essential oil components, i.e., eugenol, methyl eugenol, and β-caryophyllene in SNEDDS and ME formulations. Data are presented as mean ± SD (*n* = 3).

Eugenol exhibited good stability across all temperatures in both formulations. In SNEDDS, the percentage of eugenol remaining at day 90 was 110.51% at 4 °C, 99.49% at 30 °C, and 94.45% at 45 °C. In the ME formulation, the corresponding values were 105.31%, 104.48%, and 104.76%, respectively. Methyl eugenol also remained stable, with final concentrations of 99.25% (4 °C), 98.62% (30 °C), and 94.08% (45 °C) in SNEDDS, and 102.32%, 102.18%, and 103.53% in ME at the same time points. For β-caryophyllene, the remaining amounts in SNEDDS after 90 days were 95.75% at 4 °C, 94.92% at 30 °C, and 88.55% at 45 °C. In contrast, the ME formulation showed slightly better retention with 97.91%, 96.23%, and 94.47% remaining at 4 °C, 30 °C, and 45 °C, respectively (Figure 7).

The findings demonstrate that both SNEDDS and ME formulations can effectively preserve the chemical integrity of eugenol, methyl eugenol, and β-caryophyllene during storage under different temperature conditions. Notably, all three compounds showed excellent stability at 4 °C in both systems.

At 30 °C, the retention of all compounds remained above 94% in most cases, indicating good thermal stability under standard room-temperature storage. However, under accelerated conditions (45 °C), a modest decline in the content of the compounds was observed, particularly for β-caryophyllene in SNEDDS, which decreased to 88.55% at day 90. This reduction may reflect its higher susceptibility to oxidative degradation or volatilization in the nanoemulsified environment, although the observed values still suggest acceptable stability for practical applications [23,24].

Interestingly, the ME system tended to offer slightly better protection for all compounds at elevated temperatures compared to SNEDDS. This may be attributed to the physicochemical characteristics of the ME matrix, which could provide a more robust encapsulation mechanism or lower interfacial reactivity [25]. In contrast, SNEDDS formulations lack a true encapsulating structure in their undiluted form; the nano-sized oil droplets only form upon aqueous dispersion. This structural limitation may expose active compounds to oxidative or environmental stress before full nanoemulsion formation occurs, potentially explaining the slightly greater degradation observed. Thus, while SNEDDS offers advantages in delivery and performance, its limited capacity to shield labile constituents in the pre-dispersion state represents a formulation trade-off that must be considered in future optimization. 

### 4.7. Anesthetic Activity of Holy Basil SNEDDS and ME

The anesthetic performance of SNEDDS, ME, and ethanol-based formulations was evaluated by measuring the time to reach Stage I3 anesthesia (surgical anesthesia) and the recovery time (R1–R3) in koi carp at five concentrations (40–120 ppm).

**Figure 8:**
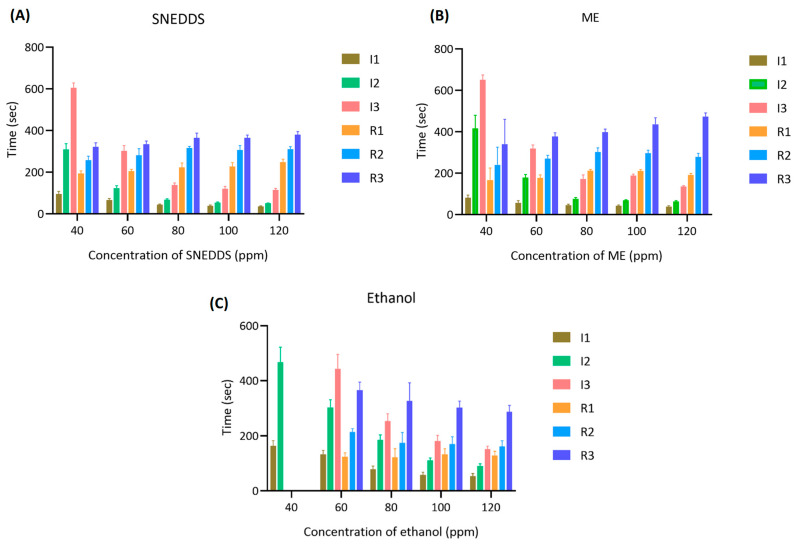
Induction time to Stage 3 anesthesia in koi carp exposed to holy basil essential oil formulated in (**A**) SNEDDS, (**B**) ME, and (**C**) ethanol at concentrations of 40, 60, 80, 100, and 120 ppm. Data are presented as mean ± SD (*n* = 10 fish per group). The findings of this study highlight the critical role of formulation type in the anesthetic efficacy of holy basil essential oil in aquatic models. Among the three tested systems, SNEDDS was the most effective at inducing surgical anesthesia (I3) rapidly and reliably across all concentrations. This can be attributed to the smaller nanometric droplet size (22.78–27.48 nm) and higher interfacial surface area of the SNEDDS system than ME, which enhances solubilization and absorption of lipophilic bioactive compounds such as eugenol and methyl eugenol. These compounds are well recognized for their central nervous system depressant and anesthetic effects in fish.

In Figure 8, at 40 ppm, only SNEDDS and ME induced Stage I3 anesthesia, with SNEDDS producing faster induction (605.70 ± 22.32 s) compared to ME (651.33 ± 23.31 s), while ethanol failed to induce surgical anesthesia in all fish (0% success). The difference in anesthetic success rates was statistically significant (*p* < 0.0001, Tukey’s multiple comparisons test) when comparing ethanol to either SNEDDS or ME. At 60 ppm, SNEDDS shortened the Stage I3 induction time to 302.90 ± 24.79 s, followed by ME (319.60 ± 16.48 s), and ethanol showed delayed induction at 443.60 ± 52.19 s. At 80 ppm, induction time improved markedly across all groups, with SNEDDS (138.60 ± 9.66 s) outperforming ME (172.40 ± 18.94 s) and ethanol (253.60 ± 25.99 s). At 100 and 120 ppm, the shortest Stage 3 induction was observed with SNEDDS (120.60 ± 11.08 s at 100 ppm and 114.70 ± 7.41 s at 120 ppm), followed by ME (188.30 ± 7.33 s and 134.90 ± 4.70 s), and ethanol (181.00 ± 20.54 s and 151.20 ± 11.75 s).

Recovery times were consistently shorter and more stable in SNEDDS formulations across all concentrations. At 120 ppm, the SNEDDS group showed the fastest average recovery (R1: 248.5 ± 14.1 s, R3: 379.60 ± 15.61 s) compared to ME (R3: 473.80 ± 16.94 s) and ethanol (R3: 286.50 ± 23.60 s). However, ethanol groups generally exhibited greater variation and irregular recovery responses, particularly at lower concentrations.

**Figure 9:**
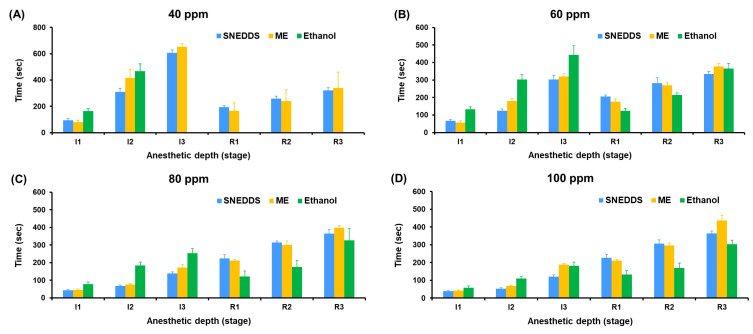
Recovery time in koi carp exposed to holy basil essential oil formulated in different concentration of SNEDDS, ME and ethanol (**A**) 40 ppm, (**B**) 60 ppm, (**C**) 80 ppm and (**D**) 100 ppm. Data are presented as mean ± SD (*n* = 10 fish per group).

To facilitate comparison of anesthetic efficacy and recovery profiles among the three formulations, Figure 9 presents a bar graph summarizing the mean induction and recovery times (±SD) across tested concentrations. This visualization highlights the superior and more consistent performance of the SNEDDS group at both onset of and recovery from anesthesia.

The ME system, while also providing nano-scale dispersion, showed slightly slower induction and longer recovery times compared to SNEDDS. This difference is likely due to the aqueous content and relatively larger droplet size of ME formulations (70.50–177.10 nm), which may reduce the rate of diffusion of active compounds across the gill epithelium. Nonetheless, ME still outperformed ethanol-based formulations in both induction efficiency and consistency.

The ethanol-based system, used as a conventional solvent control, was the least effective formulation. It failed to induce Stage 3 anesthesia at 40 ppm and required higher concentrations to produce anesthetic effects. The poor performance is likely due to the limited aqueous solubility and rapid volatility of the essential oil in ethanol, resulting in insufficient concentrations of active compounds in the water column.

Furthermore, SNEDDS not only facilitated faster induction but also exhibited relatively uniform and shorter recovery durations, which is advantageous for minimizing stress and post-anesthetic complications in aquaculture or experimental procedures. These findings underscore the superior performance of SNEDDS as a delivery system for essential oil-based anesthetics and support its further development as a safe and effective tool for fish sedation.

SNEDDS are anhydrous preconcentrates that spontaneously form fine oil-in-water nanoemulsions upon aqueous dilution. This process generates small droplets with high surface area, facilitating rapid solubilization and diffusion of lipophilic actives such as eugenol across biological membranes. However, SNEDDS lack a true encapsulating structure in their undiluted form, which may limit protection of sensitive compounds prior to dispersion.

In contrast, microemulsions (ME) are thermodynamically stable systems formed during preparation and already contain water. The presence of water and surfactant/co-surfactant systems (e.g., Tween 80, ethanol, sorbitol) allows for the formation of slightly larger droplets with more stable interfacial films. This matrix may offer better protection of volatile or labile constituents under storage but may also reduce the rate of absorption due to larger droplet size and slower diffusion kinetics.

Together, these mechanistic differences explain the superior onset and recovery performance of SNEDDS in vivo, as well as the slightly higher chemical retention observed in ME under some storage conditions. Supplementary Table S2 summarizes the physical and chemical stability data of SNEDDS and ME formulations across all tested conditions.

## 5. Conclusions

Formulating holy basil essential oil into SNEDDS and ME systems improved its stability and anesthetic efficacy in koi carp. SNEDDS showed superior physical stability, provided retention of active compounds, and possessed faster and more consistent induction and recovery times compared to ME and ethanol formulations. These results support SNEDDS as a promising natural anesthetic delivery system for use in aquaculture and potentially in clinical herbal sedation protocols.

## Data Availability

The data presented in this study are available upon request from the corresponding author. The data are not publicly available due to the privacy policy of the authors’ institution.

## Supplementary Materials

The following supporting information can be downloaded at https://www.mdpi.com/article/10.3390/pharmaceutics17080997/s1: Table S1: Changes in Particle Size (nm) of Holy Basil SNEDDS During 90-Day Storage at 4 °C, 30 °C, and 45 °C; Table S2: Summary of Stability Profiles of SNEDDS and ME Formulations Over 90 Days
