# Supplementary material for: Comparative Stability and Anesthetic Evaluation of Holy Basil Essential Oil Formulated in SNEDDS and Microemulsion Systems in Cyprinus carpio var. Koi
Source: Pharmaceutics. 2025 Jul 31;17(8):997. doi: 10.3390/pharmaceutics17080997 (PMC12389358; doi:10.3390/pharmaceutics17080997)
Supplement: Supplementary file 1 [file pharmaceutics-17-00997-s001.zip › pharmaceutics-3742495-supplementary.pdf]

Comparative Stability and Anesthetic Evaluation of Holy Basil Essential Oil Formulated in SNEDDS and Microemulsion Systems in *Cyprinus carpio* var. Koi

Table S1. Changes in Particle Size (nm) of Holy Basil SNEDDS During 90-Day Storage at 4 °C, 30 °C, and 45 °C

Day(s)	4°C	30°C	45°C
0	22.8±2.0	22.8±2.0	22.8±2.0
14	23.4±2.9	26.0±3.4	22.3±1.4
30	30.7±1.4	28.03±5.3	28.8±1.3
60	20.5±2.5	18.0±2.2	24.5±0.2
90	26.8±3.5	26.9±2.4	27.5±1.4

Table S2 Summary of Stability Profiles of SNEDDS and ME Formulations Over 90 Days

Parameter	SNEDDS	ME
Droplet Size	Stable (~23–28 nm); minor fluctuation	Significant increase (70 → ~177 nm)
PDI	Low and consistent (<0.36)	High and variable (>0.4)
Zeta Potential	Declined significantly, especially at 45 °C (−8.70 mV)	More stable (−14 to −20 mV)
Chemical stability of eugenol	98.92 - 105.69% LA	96.82 - 108.34%
Chemical stability of methyl eugenol	98.41 - 109.27% LA	95.00 - 106.91%
Chemical stability of methyl eugenol	98.41 - 109.27% LA	95.00 - 106.91%
Chemical stability of beta-caryophyllene	96.15 - 108.77%	92.79 - 103.72%
